# Doppler radar remote sensing of respiratory function

**DOI:** 10.3389/fphys.2023.1130478

**Published:** 2023-04-27

**Authors:** Mohammad Shadman Ishrak, Fulin Cai, Shekh Md Mahmudul Islam, Olga Borić-Lubecke, Teresa Wu, Victor M. Lubecke

**Affiliations:** ^1^ Department of Electrical and Computer Engineering, University of Hawaii at Manoa, Honolulu, HI, United States; ^2^ School of Computing and Augmented Intelligence, Arizona State University, Tempe, AZ, United States; ^3^ ASU-Mayo Center for Innovative Imaging, Arizona State University, Tempe, AZ, United States; ^4^ Department of Electrical and Electronic Engineering, University of Dhaka, Dhaka, Bangladesh

**Keywords:** Doppler radar, vital signs, respiratory monitoring, respiratory signature, identity authentication, data driven models, machine Learning

## Abstract

Doppler radar remote sensing of torso kinematics can provide an indirect measure of cardiopulmonary function. Motion at the human body surface due to heart and lung activity has been successfully used to characterize such measures as respiratory rate and depth, obstructive sleep apnea, and even the identity of an individual subject. For a sedentary subject, Doppler radar can track the periodic motion of the portion of the body moving as a result of the respiratory cycle as distinct from other extraneous motions that may occur, to provide a spatial temporal displacement pattern that can be combined with a mathematical model to indirectly assess quantities such as tidal volume, and paradoxical breathing. Furthermore, it has been demonstrated that even healthy respiratory function results in distinct motion patterns between individuals that vary as a function of relative time and depth measures over the body surface during the inhalation/exhalation cycle. Potentially, the biomechanics that results in different measurements between individuals can be further exploited to recognize pathology related to lung ventilation heterogeneity and other respiratory diagnostics.

## 1 Introduction

Respiratory function is a critical indicator of health yet it is often underutilized because of shortcomings in conventional monitoring methods. Even basic respiratory rate measurement can provide insight into a subject’s health and physiological stability and is one of the strongest predictors of mortality in hospitalized patients (Droitcour et al., 2009). Respiratory signatures for sedentary patients have been used to assess Sleep apnea using the apnea-hypopnea index (AHI) (Young et al., 1993). Results suggest a high prevalence of undiagnosed sleep disorders for subjects within the age range of 30–60. Sleep Disordered Breathing (SDB) has also been associated with hypertension and cancer mortality in community-based samples (Pedrosa et al., 2011; Nieto et al., 2012). Conventional methods used to assess respiratory function include measurement of *SpO*_2_ levels through pulse oximetry, respiratory expansion, and contraction through ECG-measured thoracic impedance, airflow through spirometry, thorax expansion/contraction through respiration belts, or respiratory rate through clinical observation and counting. While *SpO*_2_ levels can provide a strong indication of respiratory distress, such indications do not show up until the problem is advanced and do not provide further diagnostic indications. Measurements of the diaphragm and related muscle activity require the attachment of ECG electrodes and are subject to interference from other electrical activity in the body. Direct measurement of airflow through spirometry or chest motion through elastic belts also requires that restrictive measurement apparatus be applied to the subject’s body, and measurement of respiratory rate through observation is labor intensive and generally limited to a rough estimate. Thus, more accurate and less obtrusive means for respiration monitoring can save lives, improve quality of life, reduce hospital stays, and lower medical costs (Droitcour et al., 2009).

Measurement of cardiopulmonary function through Doppler radar measurement of thoracic displacement has been known since the 1970s (Franks et al., 1976). Infants as well as burn victims can especially benefit from contactless respiratory measurement systems. The efficacy of radar-based systems has been shown compared to other contactless approaches. It has been demonstrated that radar systems can be designed in compact monolithic packages similar to wireless telecommunications devices and can function without affecting the monitored subject (Droitcour et al., 2001; Droitcour et al., 2004; Savage et al., 2016). The range of operation for low-cost, low-power Doppler radar transceivers also makes them suitable for applications beyond healthcare including occupancy detection, search and rescue, and security applications (Park et al., 2007a; Lubecke et al., 2007; Islam et al., 2023; Song et al., 2023). Off-the-shelf radar kit designs have also been used for radar sleep monitoring and automated sleep apnea detection (Zaffaroni et al., 2009; Savage et al., 2016). The use of appropriate signal processing can isolate respiratory and heart signals accurately even when the radar system is meters or even tens of meters away from the subject (Droitcour et al., 2001; Baboli et al., 2012a). Longer-range search and rescue triage applications of Doppler radar have also emerged. The respiratory rate has been successfully monitored for a seated subject at up to 69 m, while heart rate was successfully monitored at up to 21 m, using a compact system generating tens of milliwatts of power at 2.4 GHz, similar to a cordless phone. At closer ranges associated with home and hospital patient monitoring, more detailed cardiopulmonary signatures can be analyzed using similar or lower power levels (Yamada et al., 2006). Heart rate measurements have been successfully measured at 1 m using sub-micro-watt power levels.

**FIGURE 1:**
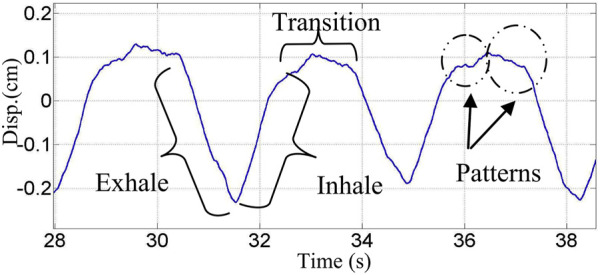
Continuous wave 2.4 GHz Doppler radar measured chest displacement. Unique signature details for inhalation, exhalation and transition regions are marked (Rahman et al., 2018).

Both heart and respiratory activity present as displacement changes at the body surface, whether the subject is standing, seated, or recumbent. Doppler radar measurement of this displacement provides a real-time signature of respiratory function which can be analyzed not only to assess the respiratory rate, but also for the assessment of respiratory volume, recognition of diagnostic patterns, and even for support in the verification of the identity of an individual (Singh et al., 2013). A signature of this type is illustrated in Figure 1, where details associated with inhalation, exhalation, and transitions provide valuable information on pulmonary function and physiological characteristics of an individual subject.

The periodic motion patterns measured by Doppler radar can be used to differentiate between normal and abnormal breathing and identify irregular episodes such as sleep apnea events (Lee et al., 2014). A sleep study was conducted for Doppler radar assessment of Obstructive Sleep Apnea (OSA) and hypopnea events. The results were compared to the gold standard polysomnography test, with accurate detection of events identifiable by a simple indicator included in the standard sleep study monitoring data. To verify the accuracy of the Doppler radar system on infants, an infant breathing simulator has been developed (Yan et al., 2009).

Alternate non-invasive biosensors, such as lasers, pressure sensors, and magnetic induction coils have been used with the same goal (Aubert et al., 1984; Reinvuo et al., 2006; Steffen and Leonhardt, 2008). Recording cardiopulmonary signals have been attempted with accelerometers directly attached to the body (Aubert et al., 1984). Using lasers requires a direct line of sight with the target. Body-attached accelerometers may have similar noise and encumbrance issues as sensors used for ECG. Infrared sensors have also been used for respiratory monitoring applications but suffer from line-of-sight requirements (Yavari et al., 2014). In comparison, Doppler radar can be used through clothing and other obstacles (Rissacher and Galy, 2015; Islam et al., 2020a).

Most non-invasive radar monitoring of respiration is focused on sedentary breathing, as it provides a consistent mechanism for comparing breathing between individuals and for tracking breathing for an individual over time. Breathing can change dramatically or even stop during certain activities. Sedentary breathing does not require knowledge of a subject’s activity but does require a mechanism for determining that sedentary conditions are met, which can be achieved by analyzing the spatial-temporal variations of the radar signal (Massagram et al., 2011). In addition to obstructive sleep apnea assessment, radar respiratory measurements of sleeping subjects have also been used to detect sleep/wake states in subjects as well as the sleep posture of a subject (Chen, 2008; de Chazal et al., 2008; de Chazal et al., 2010).

Radar-based torso displacement measurements have been used to accurately assess respiratory tidal volume which corresponds distinctly with an individual’s respiratory mechanics (Benchetrit, 2000). Accurate reconstruction of the chest wall motion implementing non-contact sensors presents the possibility of remote identification of individuals based on features of unique airflow profiles. By analyzing the unique patterns of inhalation and exhalation along with tidal volume, feature sets have been engineered to uniquely identify subjects (Rahman et al., 2016; Rahman et al., 2018; Islam et al., 2019; Islam et al., 2020b). Furthermore, unique respiratory signatures have been used to classify measurements of adults or children, and to study other mammals, as well as fish and reptiles (Hafner et al., 2012; Singh et al., 2012; Wang et al., 2019).

Notably, the standard frequencies used for radar sensing are the internationally reserved 2.4 GHz, 5.8 GHz, and 24 GHz Industrial, Scientific, and Medical (ISM) bands. Using 2.4 GHz and 5.8 GHz radars offers the use of off-the-shelf components as these are frequencies commonly used for telecommunication applications. Radar transceivers operating at 24 GHz or higher frequencies offer higher-resolution data but require more complex signal processing (Singh et al., 2013).

The Respiratory Mechanics section of this paper describes the relationship between respiratory function and torso displacement which can be measured with a radar system. The Physiological Radar section describes the principles of operation for Doppler radar motion sensing and the types of signals produced by such sensors. The Signal Analysis section describes the application of non-data-driven expert models to provide radar-based assessments of various respiratory characteristics or conditions for a subject, and also data-driven machine learning approaches to such assessments which can provide more robust analysis and can be tailored to individuals.

## 2 Respiratory mechanics

Physiological Doppler radar detects the minute movement of the chest surface due to cardiorespiratory activities including heartbeat, arterial pulsation, and breathing (Benchetrit, 2000). This physiological motion is mostly concentrated about the thorax, where the heart and lungs lie, and the abdomen (Benchetrit, 2000; Vander et al., 2020; H Opie, 2005; Lin et al., 2017). Respiratory functions for these two regions are known as thoracic and diaphragmatic breathing, respectively (Aubert et al., 1984; Lin et al., 2017). Torso deformation is a complex phenomenon based on a three-dimensional pattern that varies with the subject and activity (Vander et al., 2020; H Opie, 2005).

Biomedical Doppler radar operates at a distance and in the frequency range where most of the signal is reflected at the skin surface and thus motion sensing is primarily restricted to this region. Generally, lung contraction takes place in two ways. One is by downward and upward movement of the diaphragm to lengthen or shorten the chest cavity, and the other is by elevation and depression of the ribs to increase and decrease the diameter of the chest cavity. It has been demonstrated in various clinical investigations that a sedentary adult human subject shows diversity in respiratory patterns not only in terms of the tidal volume during inspiratory and expiratory duration but also in terms of airflow profile (Benchetrit, 2000; Vander et al., 2020; H Opie, 2005). Additionally, different people have different lung sizes, shapes, and volumes due to physiological diversity (Benchetrit, 2000; Vander et al., 2020; H Opie, 2005).

**FIGURE 2:**
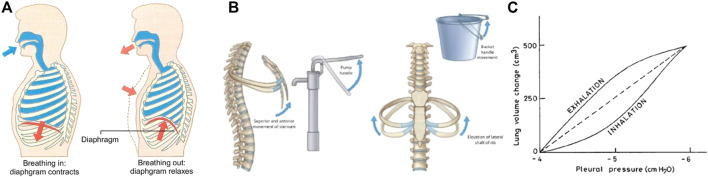
Illustration of respiratory function. Upward motion of the diaphragm during exhalation reduces lung volume and chest displacement **(A)** (Sebel et al., 1985). Displacement is also governed by sternum and rib mechanics **(B)** (Arthittayapiwat et al., 2019). Volume and corresponding displacement are not completely symmetrical and vary between subjects and depth of breathing **(C)** (Menon and Agrawal, 2003).

Chest wall movement during the airflow in the lungs is related to the contraction and relaxation of the diaphragm, as well as the external intercostal, neck, and chest muscles (Droitcour et al., 2004), as illustrated in Figure 2A. When the diaphragm pushes downward, the volume inside the lung increases and air flows into the lungs. Air continues to flow until the pressure inside the lungs equals the pressure outside the lungs, and at that point, the diaphragm relaxes and moves back. Therefore, the lung and diaphragm movement coordination are the drivers for external chest displacement, which is also controlled by the mechanics of the sternum and ribcage. Volumetric changes in the thorax correspond to sternum movement which occurs similarly to the motion of a “pump handle”, as illustrated in Figure 2B. Additionally, rib movement occurs in the lateral dimension of the thorax when the shafts are elevated, similar to the motion of a “bucket handle”, also shown in Figure 2B. The profiles for thoracic volume change during inhalation and exhalation are not completely symmetric, nor are they identical for deep and shallow breathing. Figure 2C illustrates lung compliance hysteresis for healthy breathing (Menon and Agrawal, 2003).

Thus, the displacement across the body measured by a radar system can be ultimately traced to the mechanical function of the lungs, and are also subject to the sternum, rib, and muscular dynamics of the individual. This facilitates not only the assessment of lung function through radar measurement but also the unique characterization of individual subjects.

## 3 Physiological radar

A Doppler radar motion-sensing transceiver typically transmits a continuous wave signal, receives the reflection of this signal from a moving surface, and compares the phase of the signals to demodulate the motion information (Boric-Lubecke et al., 2015). While most physiological radar measurements use a fixed-beam antenna to limit the radar field of view, beam-steering with phased array antennas has also been demonstrated to track and isolate subjects of interest.

While stationary reflecting surfaces in the radar signal path result in a constant value for the demodulated phase-difference signal, surfaces with periodic motion result in a phase demodulation output signal which varies in proportion to displacement during the displacement cycle. However, the resolution of the received signal depends on the standing wave pattern established by the nominal distance to the moving subject. Depending on the frequency of the transmitted wave, at a distance of even multiple of *λ*/4, where *λ* is the wavelength of the radar signal, destructive interference of transmitted and received signal occurs and resolution is compromised (null point). On the other hand, at a distance of every odd multiple of *λ*/4, the optimum signal level is attained (Park et al., 2007b).

### 3.1 Continuous wave doppler radar

**FIGURE 3:**
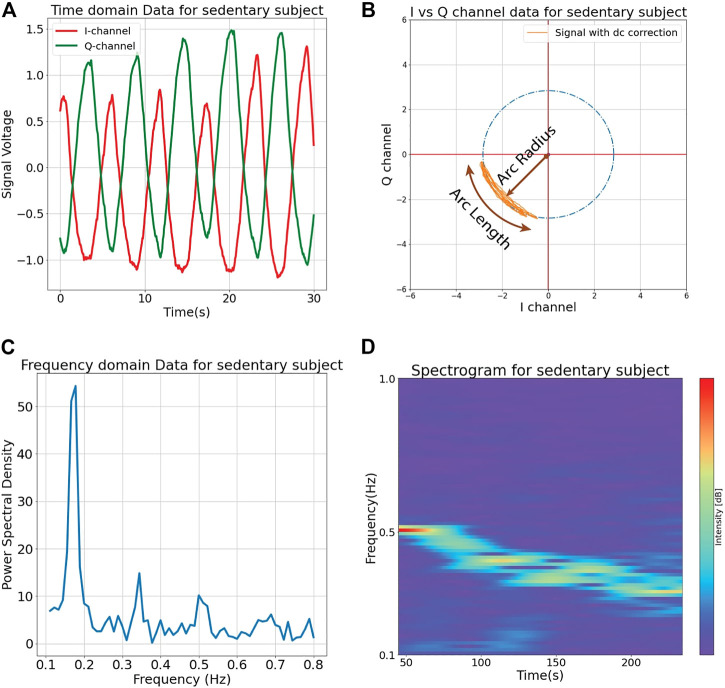
Continuous wave 2.4 GHz Doppler radar output signals measured for a sedentary subject. A quadrature receiver is used to produce two signals (I and Q) which are 90° out of phase with each other **(A)**. I and Q signals can be plotted in a complex constellation to produce an arc that describes signal strength and displacement through the radius and angular changes, respectively **(B)**. The output can also be analyzed in the frequency domain over some fixed period of time **(C)** or as a spectrogram illustrating spectral changes over time as in the decreasing respiratory rate illustrated for a subject resting after exertion **(D)**.

To manage challenges caused by this null/optimum effect various methods have been employed. One approach is the use of a continuous wave (CW) radar system which includes a quadrature receiver that uses two mixers to produce two output signals with a 90-degree offset, known as the in-phase signal (I), and the quadrature signal (Q) (Gao et al., 2012). Continuous wave systems use simple radio architecture while maintaining the advantage of fundamentally distinguishing between moving and non-moving reflecting surfaces. Stationary reflecting surfaces in the radar field of view are known as clutter, and associated reflections contribute to the radar output as a constant dc voltage shift. The time domain I and Q output signals for a quadrature radar are illustrated in Figure 3A after dc offset correction. When these two signals are plotted against each other during the period of a motion cycle (I-Q constellation), periodic displacement results in an arc with angular variation corresponding to the surface displacement and radius which varies with the received signal strength, as shown in Figure 3B. Note that a radar system operating at a higher frequency will produce a longer arc for the same displacement. A circle fitting algorithm can be used to recognize the center of the arc and thus eliminate extraneous dc offset while maintaining critical dc information in the respiratory displacement signature. Note that the arc does not exactly retrace its shape and position with each breath because of typical respiratory variation and the relationship between inhalation and exhalation mechanical function. However, updating the arc center and radius over individual respiration cycles allows useful respiratory characterization. In the time domain, the magnitude of this demodulated phase difference represents the time-varying displacement of the overall moving surface. This output signal is similar to the displacement signal that might be observed using a respiratory-belt measurement, though it contains additional temporal-spatial information that can be exploited for further analysis and insight. In the frequency domain, the fundamental frequency and related harmonics for the cyclical respiratory process can be observed, as shown in Figure 3C, or in combination with temporal variation as shown in Figure 3D. A Fast Fourier transform (FFT) is typically applied to convert discrete time-domain signatures to frequency-domain signatures. Quadrature physiological Doppler radar systems have been successfully demonstrated with the short respiratory arcs produced at 2.4 and 5.8 GHz, as well as the fully wrapped arcs produced by 24-GHz systems. Furthermore, this architecture has also been implemented for respiratory monitoring using software-defined radio technology, opening the door for incredibly flexible physiological radar systems which can be adapted to an application through software alone (Costanzo, 2019).

### 3.2 Modulated radar

Another approach involves the use of frequency-modulated continuous wave (FMCW) radar (Mostov et al., 2010; Wang et al., 2014). The frequency of the radar is cycled between a range of frequencies at a rate much faster than that of the cardiopulmonary motion. Although a null point may appear in the received signal of one of the chosen frequencies, the resolution will not be compromised at other frequencies. FMCW radar integrates phase information along with range information (Lee et al., 2019). Identification of multiple unique respiratory signatures is possible using these properties. FMCW radar requires complex transceiver systems that are highly stable as frequency changes occur over short periods of time. Ultra-wideband (UWB) radar is also a viable option to record respiratory movement (Bugaev et al., 2006). UWB radars use low-power pulses to operate and allow for range tracking. These systems generally require high-speed analog-to-digital conversion systems. Alternatively, a multifrequency radar paired with range-frequency analysis may also be used to extract comprehensive information (Soldovieri et al., 2012). Since the arc displacement is proportional to the frequency of the radar, dual-frequency radar systems may be used to analyze the general and micro properties of recorded respiratory signals with complementary information derived from arcs of different lengths (Singh et al., 2013).

For applications where spatial tracking of multiple subjects is required, FMCW and UWB radars can be advantageously used to ascertain range and direction. However, for sedentary subjects whose respiratory motion signal is of prime importance, CW radar is simpler to design and implement, and typically requires less computationally intensive signal processing. Furthermore, various methods have been used with CW radar, including beam steering, blind source separation, etc., to separately monitor cardiopulmonary vital signs for multiple subjects in the radar field of view, and to isolate and suppress sources of extraneous motion.

Apart from the more commonly used radar modes, channel state information of off-the-shelf Wifi devices has also been demonstrated to record the respiration signals of human subjects (Ishmael et al., 2022). The signal can then be analyzed using fuzzy wavelet transform and Deep Learning (DL) to yield real-time user verification (Liu et al., 2020).

While beam steering can be effective for isolating a particular subject’s cardiopulmonary motion signature, the problem becomes more complex for multiple closely-spaced subjects. Advanced techniques for the separation of signal sources include the blind source separation (BSS) algorithm which has been used to separate unique respiratory signatures in multi-subject experiments and to filter extraneous limb motion from the respiratory motion of the same subject (Vergara et al., 2008; Chen et al., 2012). Such methods demonstrate the potential to monitor the vital signs of individual subjects in a crowded observation space. Another approach to separate multiple subject respiratory signals is Independent Component Analysis (ICA) combined with Joint Approximation Diagonalization of Eigen-matrices (JADE) algorithm, which decomposes the signal to the latent unique respiratory signatures of individual subjects (Islam et al., 2020c). ICA is a technique for extracting distinct signals from a jumble of signals, while JADE involves matrix diagonalization, which has been compared favorably to other algorithms that depend on optimization procedures. The wavelet transform has also been used to successfully isolate respiratory signals reliably (Baboli et al., 2012b).

## 4 Signal analysis

Radar-measured displacement patterns can be used to characterize various cardiopulmonary functions including respiratory rate, tidal volume, and apnea/hypopnea events, based on the development of a suitable mathematical model. There have been several successful demonstrations of such measures based on models developed using domain expertise, which set generalized criteria to correlate the measurement signal with expectations for the characteristic of interest. Recently, some of these have been used as a basis for more advanced machine learning (ML) methods which use data sets to train the system to recognize and categorize measurements in subsequent data.

### 4.1 Non-data driven domain expert systems

**FIGURE 4:**
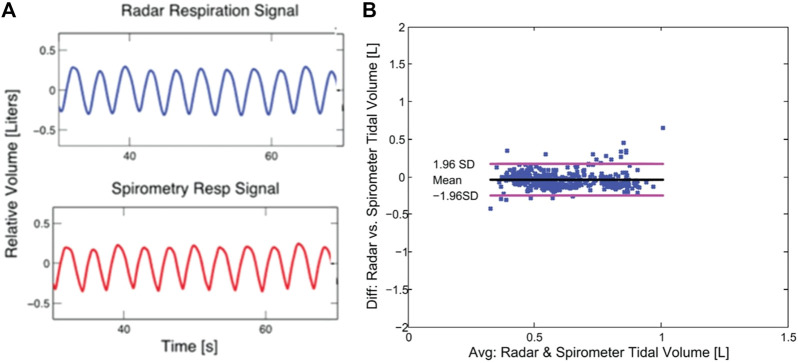
Tidal volume assessed using radar measured chest displacement. The demodulated radar time domain output matches the output of the spirometer **(A)**, and performance compared in a Bland-Altman analysis shows that the two instruments produce outputs of similar quality **(B)** (Massagram et al., 2013).

Radar measurement of respiratory tidal volume has been demonstrated for a supine subject using a linear model relating the demodulated radar-measured phase change to thorax displacement, based on an initial calibration measurement. In Figure 4, a comparison of radar and spirometer displacement plots is shown (a) along with a Bland-Altman analysis accuracy assessment (b) (Massagram et al., 2013). The findings indicate that under the appropriate circumstances, radar measurements are equivalent to those of the spirometer, without the encumbrances associated with the spirometer.

Assessments of tidal volume and other respiratory measures based on body-surface displacement patterns are subject to change when the subject does not maintain a supine posture. Through further analysis of the radar signal, it is possible to recognize changes in posture in order to properly re-calibrate measurements accordingly.

**FIGURE 5:**
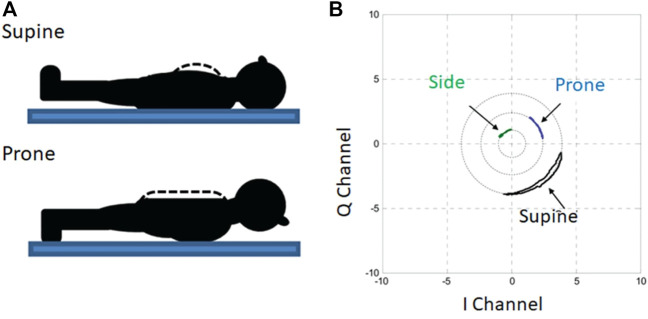
Doppler radar measurements used to assess posture of recumbent subjects. The effective radar cross section (ERCS) changes depending on the size of the body surface moving with respiration, which changes with recumbent posture **(A)**. Analysis of the I-Q plot reveals the correspondence between posture and ERCS through the plotted arc radius **(B)** (Kiriazi et al., 2021).

**FIGURE 6:**
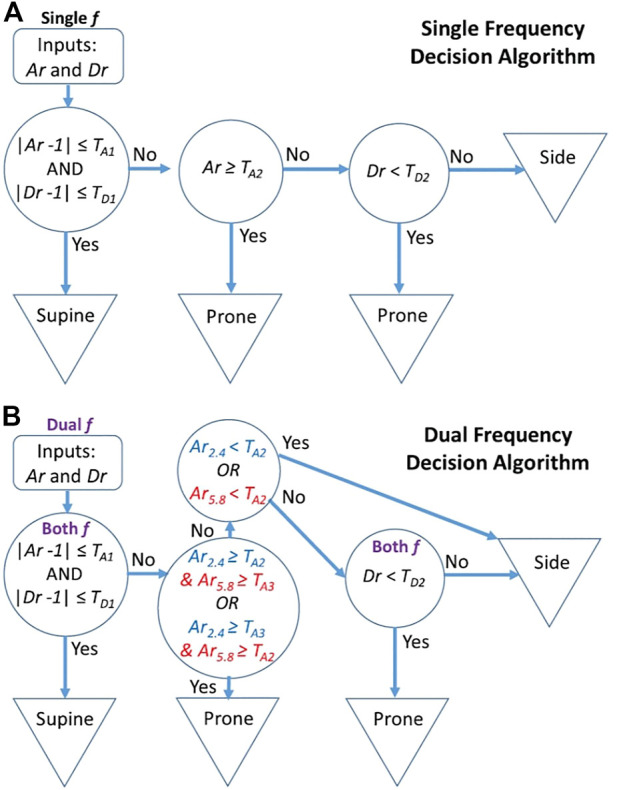
Flow charts for expert model decision algorithms for determining sleep posture from dual-frequency radar measurements. Subject sleep posture is determined based on arc radius ratio (Ar) and displacement magnitude ratio (Dr) threshold tests. The single frequency decision is based on two threshold assessments for both Ar and Dr **(A)**, while the dual frequency decision introduces a third threshold test for both Ar and Dr **(B)** (Kiriazi et al., 2021).

Kiriazi et al. designed an expert model to differentiate between supine, prone, and side postures of sleeping subjects (Kiriazi et al., 2021). Figure 5A shows the difference in the ERCS of a patient in supine and prone positions due to the variation of the total moving area facing the radar during respiration. The experiments demonstrate a significant variance between the effective radar cross section (ERCS) properties of the three sleeping positions, which relates to the moving area of the surface as shown in Figure 5B. The expert model algorithm used thresholds comparing the quadrature radar I-Q constellation arc radius (signal strength) and angle (displacement) magnitudes to identify specific sleeping postures. The process required an initial calibration in the known-supine posture and then yielded reliable tracking as the subject changed positions over time. Notably, the prone position resulted in the largest ERCS measurement, and the side posture resulted in the smallest ERCS. The flow charts for expert model decision algorithms used for determining sleep posture with a dual frequency radar sensor system are illustrated in Figure 6.

**FIGURE 7:**
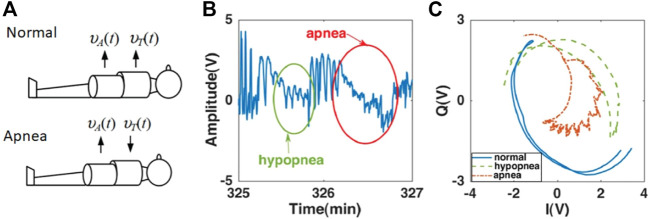
Doppler radar measurements used to recognize occurrence of obstructive sleep apnea (OSA) events. Whereas in Normal Breathing, the direction of displacement of chest and abdomen are in the same direction, the displacements are in opposite directions during Apnea Breathing **(A)** During an apnea (full respiratory obstruction) or hypopnea (partial obstruction) event, variations in chest and abdomen displacement occur which are evident in the radar time domain displacement plot **(B)**. The temporal-spatial variations caused by paradoxical breathing during apnea vents are discernible through an increase in hysteresis for the arcs traced in the I-Q plot during the respiratory cycle **(C)** (Baboli et al., 2020).

In some cases, respiration does not produce a uniform average displacement across the body. Particularly in the case of obstructive apnea, the thorax and abdomen will move out of phase with each other in a phenomenon known as paradoxical breathing, as illustrated in Figure 7A. Along with other measures, a radar system can recognize this effect and provide a corresponding diagnostic analysis. The radar-measured displacement signal is a composite of the motion across the whole body surface, and constructive and destructive interference appears in the I-Q constellation as an arc that traces back and forth through each cycle with a recognizable degree of hysteresis. An expert system model was devised from radar data for detecting an apnea or hypopnea event from the analysis of maxima and minima positions, total signal power, and breathing duration. Measurements at 2 GHz were used to readily isolate obstructive respiratory events, and higher frequency recordings at 24 GHz further facilitated the differentiation between apnea and hypopnea events, as illustrated in Figure 7.

### 4.2 Data-driven modeling system

While models using domain expertise have provided an effective starting point for diagnostic pattern recognition, inherent variations between subjects, and even for a single subject over time, present challenges. A more personally tailored method of modeling may be more effective in these cases. Machine learning has been applied for cases that demand high accuracy where features are not immediately apparent.

Machine learning (ML) methods have been applied to Doppler radar measurements used for applications including health monitoring, sleep medicine, and early detection of Alzheimer’s disease. In general, ML implementation can be separated into two categories: feature engineering to identify the salient biomarkers; and predictive modeling to identify the associations between the features and the target tasks (e.g., diagnosis). Table 1 summarizes various ML approaches which have been investigated.

**TABLE 1:** Applications of ML on Doppler radar data.

Data format	Application	Data information	Algorithms and performances	References
Time series format	Sleep Apnea Classification	4 subjects (3 male and 1 female 48 ± 6.9, 210 ± 20.5 lbs, and AHI 49 ± 29)	LDA - Acc: 73.1%	Javaid et al. (2015)
			Sen: 71.2	
			Spec:70.8%	
	Breath Patterns Classification	5 subjects (4 males and 1 female, average age 23 years)	SVM - Acc: 90%	Miao et al. (2017)
		10 subjects (7 males and 3 females)	LDA - Acc: 92.8%	Zhao et al. (2019)
			KNN - Acc: 94.4%	
			DT - Acc: 94.2%	
			SVM - Acc: 94.7%	
			Ensemble Learning: 90.0%	
	Sleep Stage Estimation	13 subjects (10 males, 3 females, 22–29 years, 53–79 kg)	KNN - Acc: 77.1%	Hong et al. (2018)
			DT - Acc: 70.2%	
			SVM - Acc: 74.2%	
			Ensemble Learning - Acc: 81.1%	
	Identity Authentication	5 subjects	KNN - Acc: 93.75	Islam et al. (2020a)
			SVM - Acc: 100%	
			RF - Acc: 91.67	
		20 subjects (16–35 years, 42–85 kg)	KNN - Acc: 86.9 SVM - Acc: 97.5	Islam et al. (2022)
	Arrhythmia Detection	15 subjects (9 males and 6 females)	MLP - Acc: 75%	Iyer et al. (2022)
Non-series format	Sleep Posture Recognition	20 subjects (14 males and 6 females 76.12 ± 12.98 kg)	SVMs with feature extracted from 2.4 GHz- Acc: 85%	Islam and Lubecke (2022)
			SVMs with feature extracted from 5.8 GHz- Acc: 80%	
			DT with features extracted from dual-frequency - Acc: 98.4%	
		20 subjects (14 males and 6 females 22–75 years, and 45.7–104.8 kg)	Rule-based Decision Algorithm- Acc:100%	Kiriazi et al. (2021)
	Breath Patterns Classification	31 subjects (20 males and 11 females, average age 25.4 years)	KNN - Acc: 99.88%	Van et al. (2019)
			DT - Acc: 96.58%	
			SVM - Acc: 99.95%	
		5 subjects (5 males, 26–31 years, and 52–76 kg)	MLP - Acc: 99%	Saeed et al. (2021)
			RF - Acc: 98%	

#### 4.2.1 Feature engineering

**FIGURE 8:**
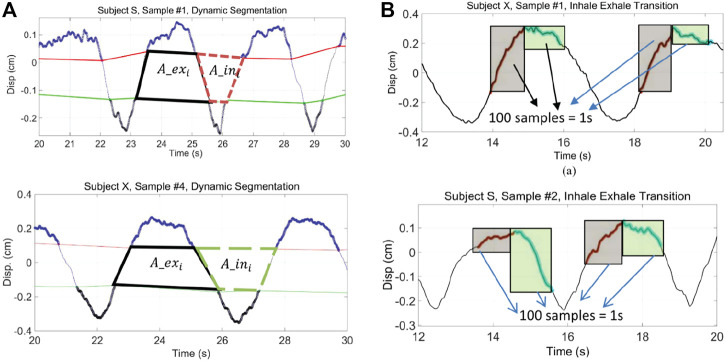
Examples of time series feature extraction for Doppler radar-based subject identity authentication. Breathing diversity is apparent for two different participants (upper vs. lower plots) as illustrated through their inhale/exhale area ratios **(A)** and variations in dynamics during the transition from inhale to exhale **(B)** (Rahman et al., 2018).

Feature engineering is used to extract a set of features based on domain knowledge from raw data. The feature vector of a sample is a characteristic description related to a target task. For Doppler radar data, human body motion, such as that from breathing and heartbeat, causes shifts in the frequency, amplitude, and phase of transmitted waves. These shifts are apparent in received signals, which can be presented in two formats for feature engineering: time series (e.g., time domain displacement) and non-series (e.g., I-Q plot, spectrogram). An example of time series radar features used for identity authentication is illustrated in Figure 8. A summary of reported feature engineering research from these two perspectives is presented here.

##### 4.2.1.1 Time series format

The output from a Doppler radar system can be presented as a time-series displacement waveform after demodulation. Often, a set of statistical measurements such as first and second-order statistics can capture the changes and variabilities in amplitude for recognizing task-related information. These measurements are applied to long- and short-term periods to obtain time-varying features (Javaid et al., 2015; Miao et al., 2017; Hong et al., 2018; Zhao et al., 2019; Islam et al., 2020a). For example, envelopes can be derived from the original time series using interpolation methods. The characteristics of the envelopes indicate the global trend of the amplitude of the time series (Javaid et al., 2015). The peaks in the upper envelope denote the transition between inspiration and expiration. In Figure 8A (Rahman et al., 2018), inhale and exhale areas can be derived from intersection areas for the time series inhale lines and exhale lines. These can be assessed with or without the inclusion of transition peaks (Islam et al., 2022). Similarly, details in the peak transitions, shown in Figure 8B can also be considered descriptive features. The statistics of the peaks may present breathing which contributes to the recognition of a breathing pattern (Miao et al., 2017; Zhao et al., 2019) and to the identity of the subject (Islam et al., 2020a). In addition to envelope-based studies, bandpass filters have been used to obtain respiration signals and heartbeat signals (Hong et al., 2018; Iyer et al., 2022). The statistics of these two signals have been used for sleep stage estimation (Hong et al., 2018). The heartbeat signal is transformed into RR intervals to derive arrhythmia-related features (Iyer et al., 2022). Features extracted from the frequency domain offer descriptions of instantaneous energy and frequency of respiration and heartbeat (Miao et al., 2017; Islam et al., 2019; Zhao et al., 2019; Islam et al., 2020a).

##### 4.2.1.2 Non-series format

The I-Q constellation has been used as an effective alternative to display the demodulated signal (Kiriazi et al., 2021; Islam and Lubecke, 2022). In the I-Q constellation (shown in Figure 3B for continuous wave 2.4 GHz Doppler radar output signals measured for a sedentary subject), the captured signal traces an arc on the complex I-Q plot. The radius and the angle scanned by the arc on the plot are proportional to the square root of the effective radar cross-section and the displacement magnitude of the moving target, respectively. The difference in cardiorespiratory activities of subjects can be jointly measured by these two factors, as in the example of sleep posture assessment. While prior work used dual-frequency radar and an expert system to accurately assess a subject’s sleep posture based on comparison with a known position calibration, machine learning has been successfully applied to make an accurate assessment using a single-frequency radar and without the need for a calibration measurement (Islam and Lubecke, 2022). In addition, time-frequency images, such as spectrograms and scalograms, illustrate the spectral density of all candidate frequencies through time. The spectral density can directly assess respiratory patterns with different rates in the corresponding frequency band (Van et al., 2019). Channel state information, originally designed to characterize interference in communications systems, has been repurposed for sensing of respiratory motion (Saeed et al., 2021; Ishmael et al., 2022).

#### 4.2.2 Predictive modeling

Extracted features are knowledge sets representing the raw data. Taking the features as the input, extensive research has been focused on developing ML models to assess the relationship between input features and outcomes. Several prevalent ML models using Doppler radar data for general applications such as subject identification and breath recognition, and medical applications such as breath disorder and sleep apnea applications are reviewed as follows.

##### 4.2.2.1 K nearest neighbor

K Nearest Neighbor (KNN) is a non-parametric classification model (Wu et al., 2007). It measures the distances between new instances to all observations based on the extracted features. For example, the distance from a new instance *x* to an observation *z* could be evaluated by the Minkowski distance of order *p*

$$dist\left(x,z\right)=\overset{d}{\underset{i=1}{\sum }}{\left(|x_{i}-z_{i}{|}^{p}\right)}^{\frac{1}{p}},$$ (1)

where *d* is the dimension of *x* and *z*, and *p* is an integer. The K nearest neighbors of *x*, then, are selected to vote for the category of each new instance. The key parameters in KNN models include the number of neighbors, K, and the associated distance measurement. KNN has shown satisfactory classification results (Hong et al., 2018; Islam et al., 2019; Van et al., 2019; Zhao et al., 2019; Kiriazi et al., 2021). Taking (Hong et al., 2018) as an example, KNN is applied to exhale and inhale features derived from Doppler radar data (see Figure 8) and achieves 95% success rate for subject identification tasks with 6 subjects. In another study with 20 subjects (Kiriazi et al., 2021), the accuracy of KNN, however, degrades to 86.9%. This indicates the performance of KNN is sensitive to the data size and quality of the dataset. It is also noted that KNN may suffer from high computational costs and memory when the instance database is large.

##### 4.2.2.2 Decision Tree

Decision Tree (DT) is a tree-structured model that progressively organizes instances into small homogeneous subsets (Hong et al., 2018). The standard DT recursively partitions instances by splitting nodes based on the information gain metric. The information gain is the expected reduction in information entropy evaluated on the classes of instances. For a set *D* with *C* classes, the information entropy, *Ent*(*D*), is calculated as:

$$Ent\left(D\right)=-\overset{C}{\underset{c=1}{\sum }}p_{c}{log}_{2}\left(p_{c}\right),$$ (2)

where *p*_*c*_ is the sample ratio of class *c*. Iterative Dichotomiser 3 (ID3) and Classification and Regression Tree (CART) are two common DT variants for binary classification and multi-class classification, respectively (Wu et al., 2007). DT has been well-adopted in medical applications due to its simplicity and ease of interpretation. In (69), DT is applied to evaluate features extracted from the two time-frequency analysis methods (short-time Fourier transform and continuous wavelet transform). The trained DT provides a tree-based decision flow to predict different levels of breathing rate. In addition, studies in (64) demonstrate that the performance of DT is comparable to other models such as KNN. However, DT is known to face challenges for continuous variables and the nature of lower model capacity. For example, in (65), the accuracy (90.3%) of DT is marginally lower compared to ML algorithm performances ( > 93.9%) for recognizing 6 breathing-disorder patterns.

##### 4.2.2.3 Linear discriminant analysis

Linear Discriminant Analysis (LDA) is originally a dimensional reduction technique to linearly project data to lower dimension space where the separability between classes is maximized (Balakrishnama and Ganapathiraju, 1998). For a binary classification task, where two classes have means *μ*_1_, *μ*_2_ and covariances Σ_1_, Σ_2_, the optimal weight *W* should be obtained by maximizing (Fisher, 1936)

$$\arg\;maxJ\left(W\right)=\frac{{\left(\mu_{1}-\mu_{2}\right)}^{2}}{W^{T}\Sigma_{1}+\Sigma_{2}W}.$$ (3)

It has been extended to multi-class classification problems. LDA determines to which regions in the lower dimension space the observation belongs, by incorporating Bayes’ theorem. LDA has been applied for different medical applications. For example, Zhao et al. (Zhao et al., 2019) study the breathing disorder problem and develop LDA as a six-class classifier to distinguish normal breathing, Cheyne-Stokes breathing, Cheyne-Stokes variant breathing, Dysrhythmic breathing, Biot’s breathing and the Kussmaul’s breathing with 93.9% accuracy. However, the performance of LDA cannot be guaranteed when the mapping relationship between feature and label is non-linear. For example, in a sleep apnea screening study (Javaid et al., 2015), 13 features are extracted from the respiratory signal and 2 are derived from the envelopes based on the hand-crafted peak finding algorithm. The classification accuracy was only 73%, which may not be considered satisfactory.

##### 4.2.2.4 Support vector machine

**FIGURE 9:**
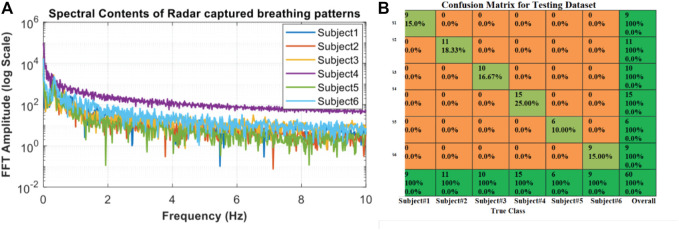
Spectral respiratory feature extraction used with SVM classification to identify six subjects. Based on the FFT for six participants **(A)**, SVM classification was used to produce 100% accurate identification of each subject, as shown in the confusion matrix **(B)** (Islam et al., 2019).

Support Vector Machine (SVM) identifies the hyper-plane that maximizes the margins between support vectors and itself (Lee et al., 2014). The support vectors are the closest observations to the hyper-plane in the feature space. This hyper-plane is the statistically optimal decision boundary to divide data into two categories. The basic SVM is empowered by kernel methods to execute hyper-plane searching in a higher-dimensional space. Kernel methods can non-linearly transform the original features into another new feature space where SVM can linearly separate these new features. The optimal hyperplane of SVM for a binary classification task is:

$$f\left(x\right)=\overset{N}{\underset{i=1}{\sum }}a_{i}y_{i}K\left(x_{i},x\right)+b,$$ (4)

where *N* is the number of samples and *a*_*i*_ is the Lagrange multiplier of *ith* sample. The kernel function is denoted by K and b could be further determined by using the support vectors. In general, SVM has shown strong learning ability for various tasks (Miao et al., 2017; Hong et al., 2018; Islam et al., 2019; Van et al., 2019; Zhao et al., 2019; Islam et al., 2020a; Kiriazi et al., 2021; Islam et al., 2022), where the selection of the kernel is crucial. For example, an FFT-based feature extraction approach with an SVM classification algorithm has been used to uniquely identify six different participants based on their normal captured breathing patterns (Islam et al., 2019). Figure 9A shows a plotted graph of the spectral contents of the participants’ respiratory patterns. The spectral properties were used to train the classifier and generate a confusion matrix, as shown in Figure 9B, leading to 100% recognition of all six participants. However, the complexity of SVM training is highly dependent on the size of the dataset. Taking the task of sleep-stage estimation as an example, SVM is applied to make predictions minute by minute for hours of sleep recordings. The number of samples becomes large and also the SVM’s performance degrades to 74.2% in terms of accuracy in (64). In addition, compared to the ensemble model, the model capacity of SVM could limit its performance for a large dataset.

##### 4.2.2.5 Ensemble learning

Ensemble learning aims at combining prediction results from multiple base learners to achieve accurate and robust performance. Ensemble learning methods usually are divided into bagging and boosting. The bagging method arranges for several base learners to independently learn knowledge mappings whereas the base learners of the boosting method sequentially learn based on the learning result of the last learner. One example ensemble method is Random Forest (RF) which consists of multiple DTs contributing to the final prediction result (Breiman, 2001). Compared to a single model, Ensemble learning methods usually reduce bias/variance but increase computational complexity (Franks et al., 1976). For breathing disorder recognition, ensemble learning has been used to eliminate imperfection for a single classifier, i.e., KNN, DT, and LDA, and is the best performer among candidates in terms of accuracy, 94.7% (Zhao et al., 2019). The bagged tree boosted tree, and subspace KNN, which are ensemble models, show superiority for sleep stage classification due to their larger model capacities for knowledge learning (Hong et al., 2018).

##### 4.2.2.6 Multilayer perceptron

Multi-layer perceptron (MLP) executes a linear mapping to project input information and then makes a non-linear activation for valuable information in each layer (McCulloch and Pitts, 1943). The parameters for the linear mapping are optimized by the gradient backpropagation from the loss function, which evaluates the difference between prediction and ground truth (Lillicrap et al., 2020). Iteratively, an *L*-layers MLP is comprised of *L* sets of learnable parameters. For each set, the parameters, *w*, and *b*, are the weight and bias parameters respectively for linearly mapping the input to the output. In the *l*th layer, these parameters project and transform the input *x* as follows:

$$f_{l}\left(x\right)=\sigma \left(w_{l}x+b_{l}\right),$$ (5)

where *σ* is the non-linear activation function. There are multiple activation functions available for modeling, such as sigmoid, tanh, relu, leaky relu, and so on. MLP is a connectionism method and brings flexibility and effectiveness for modeling the mapping between features of CSI and respiratory patterns (Saeed et al., 2021). In this study, the accuracy of MLP for six types of respiratory patterns is 99%, which is higher than 98% by RF. It is the basic version basis of the deepest learning (DL) methods, such as autoencoder, convolutional neural network, and recurrent neural network. Different from the traditional ML methods, DL methods embed feature engineering in the training process. The processes of feature engineering and knowledge mapping are conducted in an end-to-end manner (LeCun et al., 2015). DL models also show great potential for pattern recognition in Doppler radar sensing applications (Sakamoto et al., 2018).

## 5 Conclusion

Doppler radar has been successfully used to accurately assess various human respiratory characteristics. These include general measurements such as respiratory rate and tidal volume, as well as interpretive analysis including sleep apnea event detection and unique subject identification. Radar systems can produce multi-dimensional representations of body surface motion which can be analyzed through intuitive mathematical models associating mechanical motion with respiratory function, or through machine learning approaches that use data sets for known conditions of interest to create models that can be used to accurately recognize those conditions in subsequent data. Through further research, correlations may be drawn between additional respiratory health and function and measurable radar signatures to advance this non-invasive approach to broader analytics including assessment of lung ventilation heterogeneity and other diagnostically significant conditions.
